# Radium-223 for primary bone metastases in patients with hormone-sensitive prostate cancer after radical prostatectomy

**DOI:** 10.18632/oncotarget.17311

**Published:** 2017-04-21

**Authors:** Vera Wenter, Annika Herlemann, Wolfgang P. Fendler, Harun Ilhan, Natalia Tirichter, Peter Bartenstein, Christian G. Stief, Christian la Fougère, Nathalie L. Albert, Axel Rominger, Christian Gratzke

**Affiliations:** ^1^ Department of Nuclear Medicine, Ludwig-Maximilians-University of Munich, Munich, Germany; ^2^ Department of Urology, Ludwig-Maximilians-University of Munich, Munich, Germany; ^3^ Comprehensive Cancer Center, Ludwig-Maximilians-University of Munich, Munich, Germany; ^4^ Department of Nuclear Medicine and Clinical Molecular Imaging, Eberhard Karls University, Tuebingen, Germany; ^5^ German Cancer Consortium, German Cancer Research Center Partner Site Tuebingen, Tuebingen, Germany

**Keywords:** hormone-sensitive prostate cancer, radium-223, radical prostatectomy, bone metastases, off-label use

## Abstract

Radium-223 dichloride (Ra-223) is the first bone-targeting agent showing improvement in overall survival in patients with castration-resistant prostate cancer (CRPC) and bone metastases. We aimed to assess feasibility of Ra-223 treatment in patients with metastatic hormone-sensitive prostate cancer (mHSPC). Ten patients with primary bone metastases received Ra-223 following radical prostatectomy (RP). Changes in alkaline phosphatase (ALP) and prostate-specific antigen (PSA) were recorded, while pain intensity was evaluated using the self-reporting Brief Pain Inventory (BPI) questionnaire. Bone scintigraphy (BS) was performed to assess treatment response. Seven patients completed six cycles of Ra-223. Discontinuation was due to leuko- and lymphopenia, progressive lymph node metastasis or newly diagnosed liver metastasis. Treatment-related adverse events occurred in three patients and included leuko- and lymphopenia, fatigue, abdominal discomfort and nausea. Overall, a median decrease of 28% in ALP and a median decrease of 83% in PSA were noted at follow-up. However, PSA progressed in five patients at follow-up. Improvement of pain was observed in all patients (median decrease of 36% after 3 cycles and of 40% at the end of therapy). On BS, three patients showed remission, four had stable disease, and one showed progressive disease at follow-up. Our results suggest that Ra-223 for primary bone metastases in patients with mHSPC after RP is feasible and alleviates pain. ALP, rather than PSA, may be a good marker for assessing treatment response. Ra-223 could therefore be taken into consideration as part of a multimodal approach for carefully selected patients with advanced prostate cancer.

## INTRODUCTION

In patients with metastatic castration-resistant prostate cancer (mCRPC), bone is a common metastatic site, with 33% of patients developing bone metastases at two years [1]. Overall, up to 90% of patients with mCRPC will show radiological evidence of bone metastases [2], which may lead to skeletal-related events (SREs) [3]. SREs are significantly affecting the patient's mobility, functional status and quality of life (QOL) [4], and may initially reduce survival in these patients [5]. Without any treatment, approximately 50% of patients with mCRPC and bone metastases will experience at least one SRE during a 24-month follow-up period [6].

Radium-223 dichloride (Ra-223) is a targeted alpha-emitter which acts as a calcium mimetic by forming complexes with the bone mineral hydroxyapatite [7]. It selectively accumulates in areas of bone with high turnover (eg, bone metastases) and emits heavy alpha-particles that have a short penetration range with less than 100 μm [7, 8]. To date, Ra-223 is the first bone-targeting agent to demonstrate improved overall survival in patients with CRPC and bone metastases, in addition to palliating bone pain, improvement in QOL, and prolonging time to SREs at the same time [9].

While the combination of chemotherapy and hormone therapy has shown to significantly improve survival in metastatic hormone-sensitive prostate cancer (mHSPC) patients in recent randomized trials [10, 11], not all patients qualify or are willing to undergo chemotherapy. Cytoreductive surgery for mHSPC patients is an emerging area of interest [12, 13]. Potential benefits include local control, delayed initiation of androgen deprivation therapy (ADT), and possibly improved oncologic outcome [14]. Currently, multiple trials are ongoing to assess the benefits of radical prostatectomy (RP) in mHSPC patients with bone metastases as part of multimodal treatment.

In this present study, we aimed to evaluate the feasibility of off-label Ra-223 treatment in mHSPC patients with primary bone metastases after RP.

## RESULTS

### Patient characteristics and Ra-223 treatment

Patient characteristics and Ra-223 treatment are summarized in Table 1 and 2, respectively. Mean patient age was 67 ± 12.4 years. All patients were diagnosed with extraprostatic, high-grade disease at RP specimen and had radiological or scintigraphic evidence of bone metastases (cM1b) at presentation (Table 1). On average, Ra-223 treatment was started 22.2 ± 32.0 months after initial diagnosis of prostate cancer (PCa) and 7.9 ± 9.9 months after RP. Seven patients completed all six cycles of Ra-223 (mean cumulative dose 24.6 ± 4.9 MBq). Treatment-related adverse events were observed in three patients (patients 3, 4 and 7). Fatigue was reported in two patients (patients 4 and 7). Patient 7 additionally reported mild abdominal discomfort and nausea. Fatigue, abdominal discomfort and nausea did not affect continuation of therapy. Overall changes in blood counts are presented in Table 3. We found slight to moderate decreases - which did not affect continuation of Ra-223 treatment (except in patient 3) - in erythrocytes and lymphocytes in all patients, in hemoglobin, leukocytes and neutrophils in nine patients, and in thrombocytes in eight patients. In patient 3, neutrophils decreased from 2.46 G/l to 1.52 G/l (Common Terminology Criteria for Adverse Events (CTCAE) Grade I) and lymphocytes decreased from 1.3 G/l to 0.55 G/l (CTCAE Grade II) leading to discontinuation of treatment after cycle 3. In the two other patients, Ra-223 treatment was discontinued due to newly diagnosed liver metastasis (patient 9), or to progressive pelvic lymphadenopathy after cycle 2, resulting in lymphedema of the lower extremities and radiotherapy was initiated (patient 10).

**Table 1:** Patient characteristics

Patient	1	2	3	4	5	6	7	8	9	10
**Age (yr)**	79	55	77	76	67	53	60	71	46	82
**Date of RP**	Mar 2014	Mar 2014	Mar 2015	Feb 2014	Jun 2013	Oct 2013	Sep 2012	Aug 2013	Oct 2013	Nov 2014
**Histopathology**	pT3b pN1 (1/24) cM1b R1	pT3b pN1 (4/8) cM1b R0	pT3b pN0 (0/27) cM1b R1	pT3b pN1 (6/6) cM1b R1	pT3b pN1 (1/9) cM1b R1	pT3b pN1 (4/18) cM1b R1	pT3a pN0 (0/21) cM1b R0	pT3b pN1 (2/11) cM1b R1	pT3b pN1 (5/29) cM1b R1	pT3b pN1 (1/4) cM1b R1
**Gleason score**	9 (5+4)	8 (5+3)	9 (4+5)	9 (4+5)	10 (5+5)	9 (4+5)	9 (4+5)	9 (5+4)	10 (5+5)	9 (5+4)
**PSA at diagnosis (ng/mL)**	4.6	186	694	117	43.5	289	17	7278	18.4	23

RP = Radical prostatectomy; PSA = Prostate-specific antigen.

**Table 2:** Ra-223 treatment summary

Patient	1	2	3	4	5	6	7	8	9	10
1^st^ cycle Ra-223	Jun 2014	May 2014	Mar 2015	Jul 2014	Dec 2013	Aug 2014	Jul 2015	Jan 2014	Dec 2013	Nov 2015
No. of cycles (cumulative activity in MBq)	6 (21,90 MBq)	6 (22,12 MBq)	3 (10,41 MBq)	6 (18,64 MBq)	6 (27,49 MBq)	6 (30,67 MBq)	6 (30,54 MBq)	6 (21,13 MBq)	3 (18,96 MBq)	2 (7,40 MBq)
Early discontinuation	No	No	Yes	No	No	No	No	No	Yes	Yes
Reason of discontinuation	−	−	Neutropenia, lymphopenia	−	−	−	−	−	Liver metastasis	Progressive lymph node metastasis
Ra-223-related adverse events	−	−	Neutropenia, lymphopenia	Fatigue	−	−	Fatigue, nausea, abdominal discomfort	−	−	−
ALP (U/l) at 1^st^ cycle Ra-223	173	52	159	63	360	92	108	79	73	85
PSA (ng/mL) at 1^st^ cycle Ra-223	0.11	0.81	529	2.21	0.64	89	0.16	30	3.2	0.8
Concomitant medical treatment	Bicalutamide, LHRH-A	Bicalutamide, LHRH-A	Bicalutamide, LHRH-A	LHRH-A	LHRH-A	Bicalutamide, LHRH-A	Bicalutamide, LHRH-A	Bicalutamide, LHRH-A	Bicalutamide, LHRH-A	LHRH-A
ALP response (baseline → follow-up)	SD	SD	SD	SD	PR	PR	PR	PR	SD	SD
Response on BS (EOD baseline → EOD follow-up)	PD (1→2)	SD (3→3)	SD (3→3)	PR (3→1)	PR (3→2)	SD (3→3)	SD (1→1)	PR (2→1)	−	−
										
										

**EOD grade classification (modified according to Takahashi et al., 2012 [19])**: 0 - normal or abnormal findings due to benign bone disease; 1 - ≤6 bone metastases, (each of which is ≤50% of the size of the size of a vertebral body); 2 - between 6 and 20 bone metastases; 3 - >20 bone metastases but less than or equal to that on a ‘‘super bone scan’’; 4 - ‘‘super bone scan’’ or its equivalent (eg, metastasis to >75% of the ribs, vertebrae, and pelvic bones)

Ra-223 = Radium-223 dichloride; MBq = Megabecquerel; PSA = Prostate-specific antigen; LHRH-A = Luteinizing hormone-releasing hormone agonist; ALP = Alkaline phosphatase; BS = Bone scintigraphy; EOD = Extent of disease; SD = Stable disease; PR = Partial remission; PD = Progressive disease

**Table 3:** Change in blood counts during Ra-223 treatment

change	Hemoglobin		Erythrocytes		Leukocytes		Thrombocytes		Neutrophils		Lymphocytes	
	absolute change(g/dL)	relativechange(%)	absolute change(T/L)	relative change(%)	absolute change(g/L)	relative change(%)	absolute change(g/L)	relative change(%)	absolute change(g/L)	relative change(%)	absolute change(g/L)	relative change(%)
**Mean**	−0.90	−6.95	−0.43	−10.26	−1.08	−19.75	−22.20	−9.41	−0.54	−15.50	−0.37	−27.77
**Median**	−0.85	−6.58	−0.51	−12.03	−1.17	−18.60	−11.00	−4.68	−0.68	−19.45	−0.37	−28.11
**SD**	0.68	4.94	0.24	5.56	1.09	19.42	27.70	13.19	1.03	26.64	0.20	14.45
**Min**	−1.90	−13.97	−0.72	−15.71	−2.30	−51.11	−68.00	−37.78	−1.63	−46.84	−0.75	−57.69
**Max**	0.20	1.43	−0.02	−0.43	1.60	23.53	13.00	7.00	2.06	49.76	−0.10	−6.94

Baseline values before cycle 1 were compared with lowest values during follow-up. Absolute and relative changes are presented. On average, a slight to moderate decrease was noted for all cell lines.

SD = Standard deviation; Min = Minimum; Max = Maximum.

### Changes in ALP and PSA

**Figure 1:**
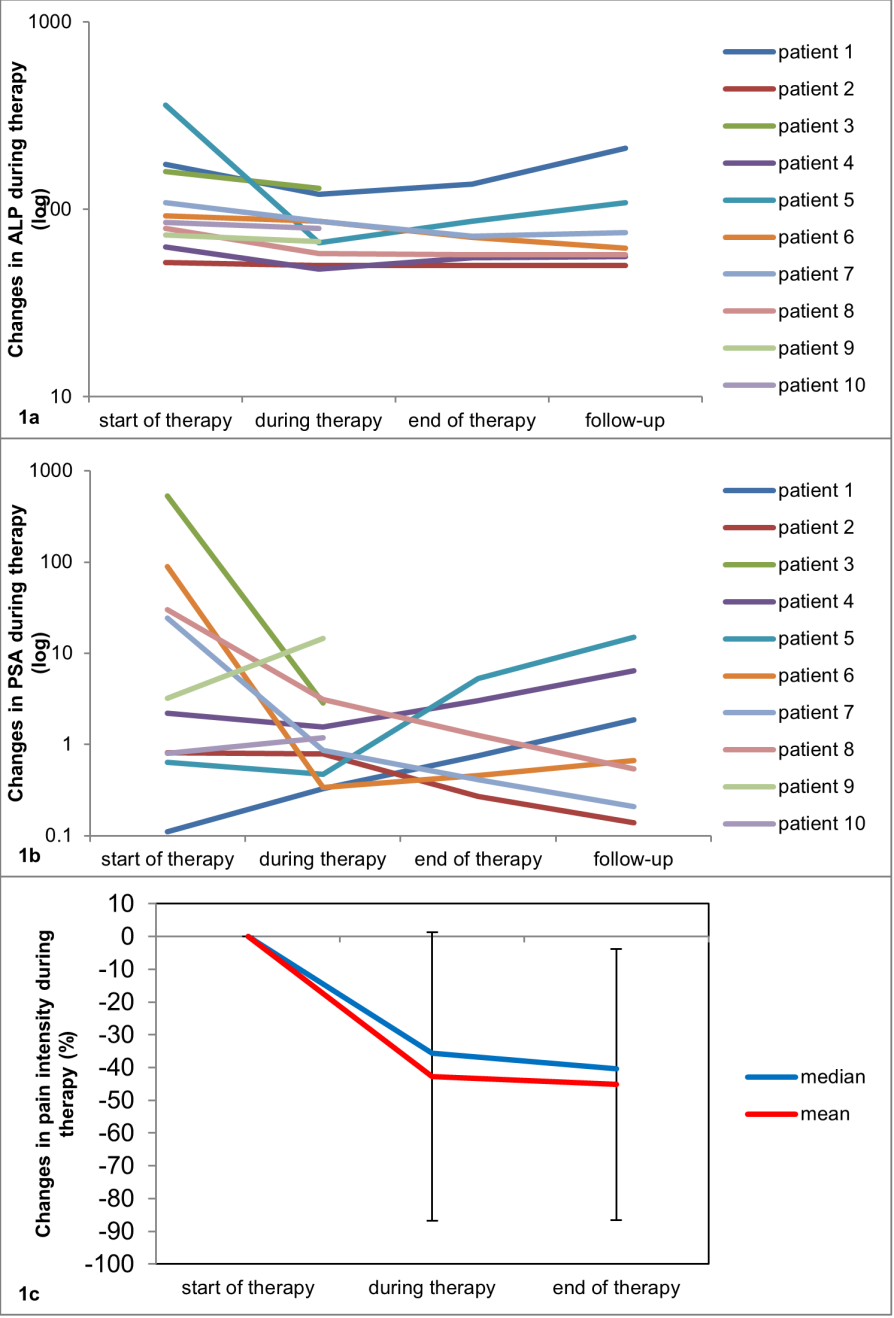
**(a-c)** Changes in ALP **(a)**, PSA **(b)**, and pain intensity **(c)** during Ra-223 therapy. The changes in ALP and PSA values are presented during therapy (before cycle 4), at the end of Ra-223 therapy (before cycle 6) and during follow-up (up to 8 weeks after cycle 6) compared to baseline values before cycle 1. The mean pain intensity decreased by 43 ± 44% during therapy (before cycle 4) and by 45 ± 41% at the end of treatment compared to baseline values before cycle 1.

Baseline alkaline phosphatase (ALP) level varied from 52 to 360 U/l (mean ALP 123.3 ± 101.2 U/l) prior to first Ra-223 administration. Individual changes in ALP values are shown in Figure 1a. Mean and median ALP decreased by 22 ± 29% and by 28% at follow-up compared to baseline. At follow-up or after discontinuation of Ra-223 treatment, partial response (decrease in ALP between 25-75%) was seen in four patients (patients 5-8) and stable disease (ALP change from -25% to +25%) in six patients (patients 1-4 and 9-10). Complete response in ALP (decrease in ALP >75%) and progression (increase in ALP >25%) were not observed. Overall, nine patients had a decrease in ALP at follow-up. One patient (patient 1) with progressive disease on bone scintigraphy (BS) presented with an ALP increase of 23%. Decrease in ALP was more evident in patients with partial remission (mean decrease 36 ± 30%) compared with patients with stable disease on BS (mean decrease 21 ± 13%). Baseline prostate-specific antigen (PSA) values varied from 0.11 to 529 ng/mL (mean PSA 75.5 ± 172.5 ng/mL). Individual changes in PSA values are shown in Figure 1b. Mean PSA increased by 523 ± 981% (median PSA decreased by 83%) at follow-up compared to baseline. At follow-up or after discontinuation of Ra-223 treatment, good response in PSA (decrease in PSA >75%) was seen in five patients (patients 2, 3 and 6-8), and PSA progression (increase in PSA >25%) in five patients (patients 1, 4, 5, 9, 10).

### Pain scores

Improvement of bone pain was observed in all patients. Mean pain intensity assessed by self-reporting Brief Pain Inventory (BPI) questionnaire [15], decreased by 43 ± 44% after 3 cycles of Ra-223 and by 45 ± 41% at the end of treatment compared to baseline (Figure 1c).

### Bone metastases

**Figure 2:**
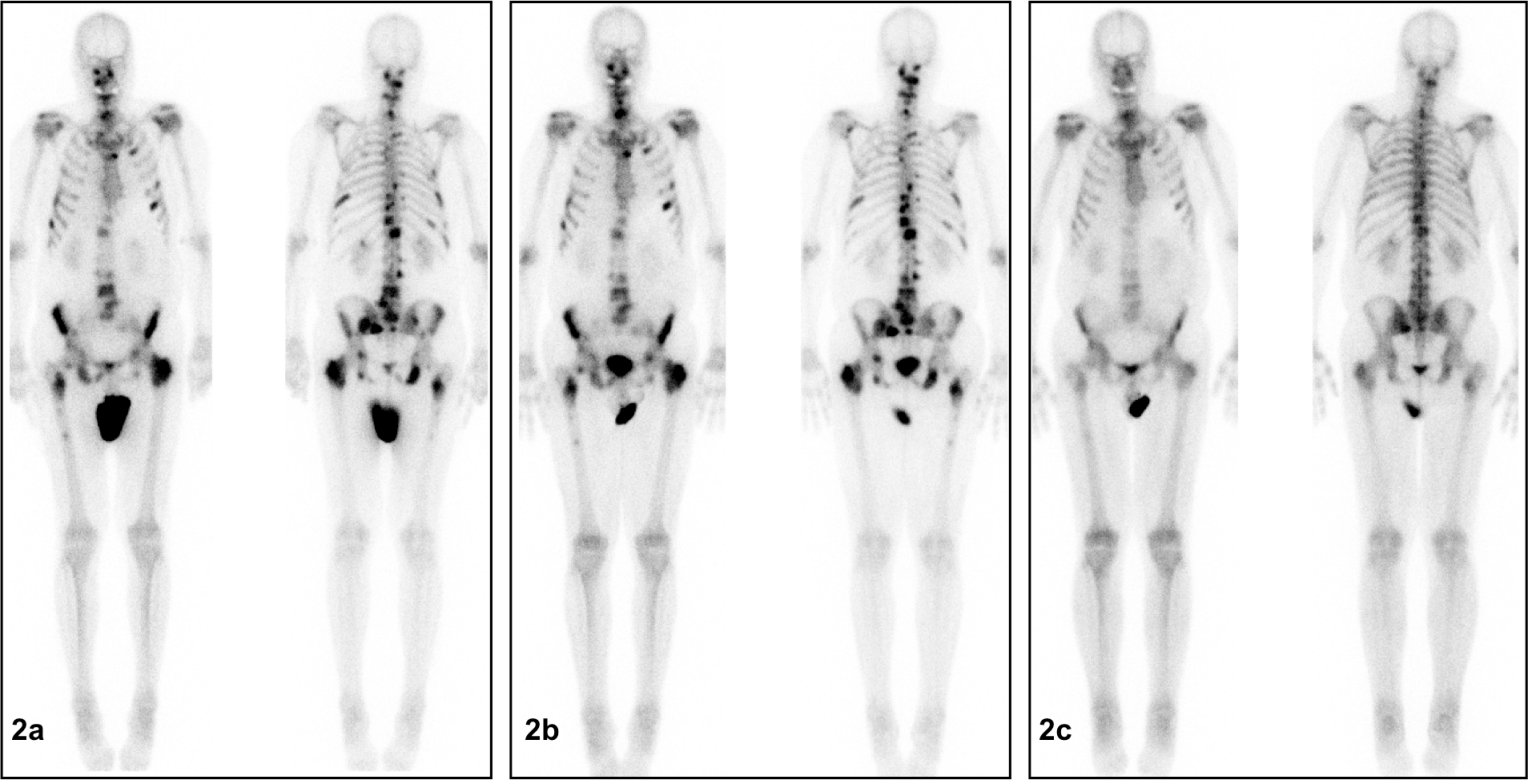
**(a-c)** Significant disease regression on bone scintigraphy (BS) after completion of Ra-223 treatment (patient 5). **(a)** (left): BS before start of therapy (before cycle 1): Evidence of disseminated bone metastasis of vertebrae, rips, pelvis and femora. **(b)** (center): BS during therapy (between cycles 3 and 4): No significant change from baseline. **(c)** (right): BS at follow-up visit: Significant decrease of tracer uptake in disseminated bone metastasis of the vertebrae, rips, pelvis and the femora compared to baseline BS. No evidence of new bone lesions.

**Figure 3:**
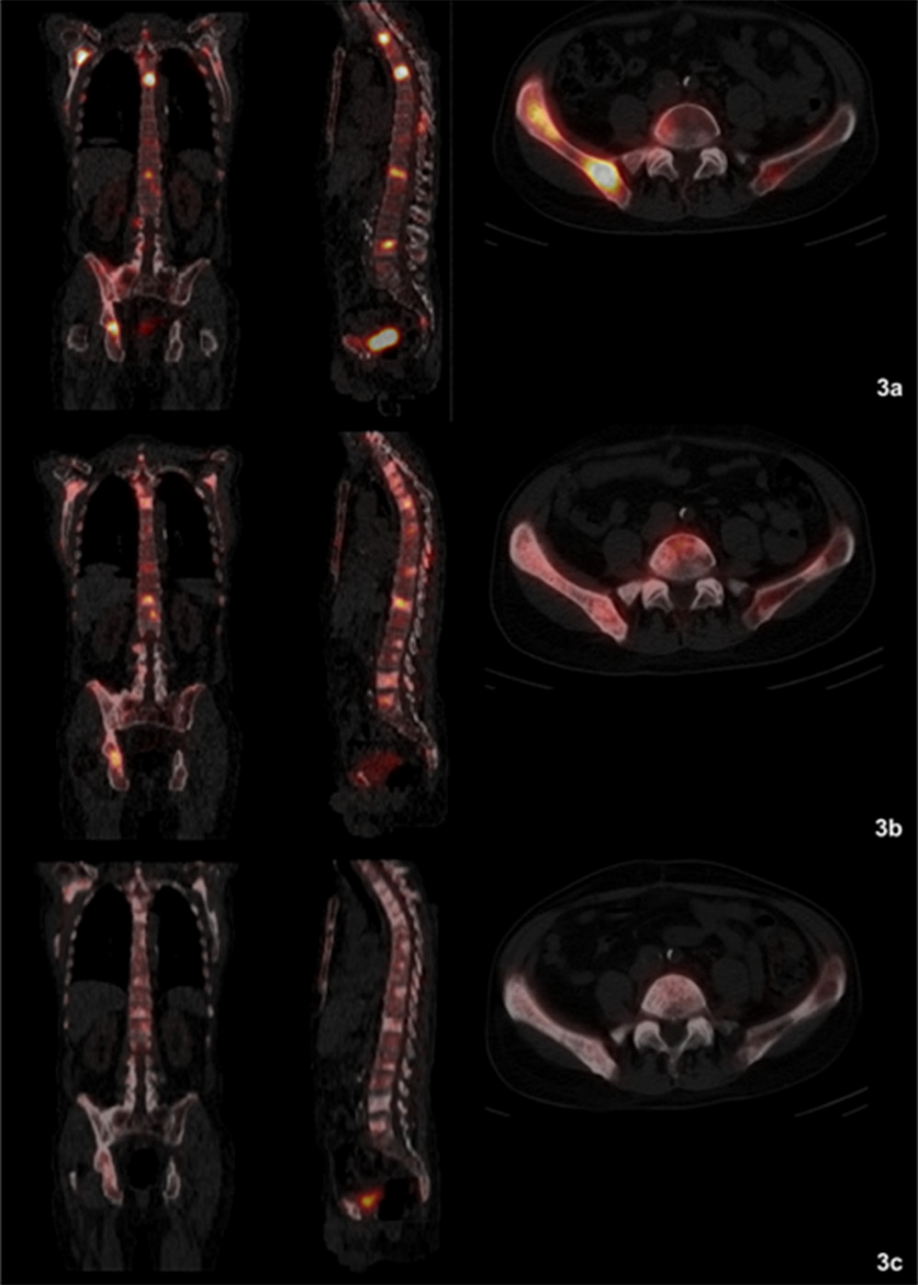
**(a-c)** Fused Single Photon Emission Computed Tomography (SPECT)/CT images clearly depicting a decreasing radionuclide uptake in the bone metastases (patient 4). **(a)** (top row): SPECT/CT before start of therapy (before cycle 1): Evidence of disseminated bone metastasis with high radionuclide uptake. **(b)** (middle row): SPECT/CT during therapy (between cycles 3 and 4): Decreasing uptake in bone metastases. **(c)** (bottom row): SPECT/CT at follow-up visit: Significantly decreasing and missing radionuclide uptake in bone metastases compared to baseline images. No evidence of new bone lesions.

Three patients responded well to Ra-223 treatment with a decrease in extent of the disease (EOD) score (partial remission; patients 4, 5, and 8). In two of these patients (patients 4 and 5) ≥1 bone lesion was not visible anymore on BS during follow-up visit (Figures 2a-2c, 3a-3c). In four patients stable disease was documented after six cycles of Ra-223 (patients 2, 6 and 7), or after three cycles of Ra-223 (patient 3), respectively. Furthermore, progress of disease with an increase in EOD score was noticed in patient 1. In two patients (patients 9 and 10) BS was not performed due to early discontinuation of Ra-223 treatment.

## DISCUSSION

In patients with newly diagnosed mHSPC chemohormonal therapy leads to significantly improved survival [10, 11]. However, not all patients qualify for chemotherapy. Cytoreductive surgery may represent an additional option. Recent trials showed that RP can be performed safely in selected patients with mHSPC [13]; in addition, recent series showed significant survival benefits in patients with mHSPC after RP [12, 16]. However, these studies are limited by their retrospective nature and potential selection bias. Randomized controlled trials are needed and currently ongoing in Europe and the US (e.g. NCT02454543, NCT01751438).

In a patient with systemic disease it is evident that RP can only be one step in a multimodal approach. Complications of co-existing bone metastases in both castration-resistant and hormone-sensitive PCa patients, such as pain or SREs, may have significant impact on their daily life [4]. The alpha-emitter Ra-223 is the first agent delaying the onset of these complications and to improve quality of life and to prolong overall survival [9]. Yet, Ra-223 has only been approved in patients with mCRPC. To date, there are no data available with regard to Ra-223 treatment for primary bone metastases in patients with mHSPC after RP. Thus, the aim of our study was to test the feasibility of Ra-223 treatment in this specific patient cohort.

Treatment-related adverse events in our study were manageable and similar to those recorded in the ALSYMPCA trial [9]. Fatigue was the most common adverse event in our patient cohort (2 of 10 patients), which was reported in 26% of patients in the Ra-223 group by Parker et al. [9, 17]. In our study, three patients did not complete all six cycles of Ra-233. Discontinuation was due to leuko- and lymphopenia, newly diagnosed liver metastasis, or progressive lymph node metastasis. In patient 3, neutrophils were low, but still in normal range prior to first Ra-223-treatment. After initiation of Ra-223 a significant decrease in neutrophils was observed prompting us to discontinue treatment in this patient. The newly diagnosed liver metastasis in patient 10 during Ra-223 treatment indicated the aggressiveness of his disease, leading to his early death approximately nine months after RP. Patient 10 showed progressive pelvic lymphadenopathy on computed tomography (CT), resulting in lymphedema of the lower extremities. As a result, treatment was interrupted after cycle 2 and radiotherapy was initiated. In this patient, only four lymph nodes were removed during RP, which was due to massive adhesions caused by former bilateral open hernia repairs with surgical mesh implantation. More extensive pelvic lymph node dissection (pLND) might have prevented progressive lymph node metastasis in this patient and thus, Ra-223 treatment might have not been discontinued.

Complete response in PSA was seen in five patients; the other 50% of patients showed an increasing PSA at follow-up when compared to baseline PSA. Increases in PSA levels might be explained by new occurrence or progression of lymph node metastasis or visceral metastasis at the time of therapy. In our study, CT or positron emission tomography (PET)/CT scan to evaluate extraosseous metastases were not performed on a regular basis. On the other hand, in some patients ADT was started shortly before the first Ra-223 administration and thus, decrease in PSA is partly due to this effect. In a previous study, PSA levels did not correlate well with findings on BS [18]. Therefore, PSA might not be the ideal marker for evaluating response to Ra-223 treatment. ALP instead might be a better marker for evaluating response to Ra-223 treatment. An increase in ALP was only seen in one patient (patient 1). This increase correlated with the patient's progressive disease on BS.

Ra-223 therapy was associated with pain relief in our patient cohort. This finding is in line with results from the ALSYMPCA trial, in which patients reported pain reduction following Ra-223 treatment [9]. According to EOD classification [19, 20], Ra-223 treatment showed partial remission in three of eight patients, and stable disease in four of eight patients on bone scintigraphy. However, the clinical value of bone scintigraphy response to Ra-223 treatment has not been validated so far.

These promising findings did not result in a general change of treatment decisions at our academic center. Generally, our approach in M1 PCa patients with symptomatic bone metastases and lymph node disease at time of diagnosis is still chemohormonal therapy, stratified by high- or low-volume disease. However, selected men are now offered RP with cytoreductive intent followed by Ra-223 treatment if chemotherapy is contraindicated as experimental approach.

Limitations to our study include the heterogeneous patient population, the small cohort size and the retrospective design. Reimbursement for the off-label Ra-223 treatment was provided by the patient's health insurance after individual application, creating a possible selection bias. The effect of ADT in combination with RP and Ra-223 is unclear; however, considering the mechanism of action of both ADT and Ra-223 a synergistic effect can be postulated. Given the short follow-up, no information on survival could be obtained.

Despite these limitations, this study represents the first case series investigating off-label use of Ra-223 for primary bone metastases in patients with mHSPC after RP and therefore may influence the design of future randomized trials. The concomitant use of Ra-223 with abiraterone or enzalutamide in patients with mHSPC may also be possible - a treatment combination that has already been shown to be safe in mCRPC patients with bone metastases [21].

Our results suggest that off-label use of Ra-223 for primary bone metastases in patients with hormone-sensitive PCa after RP is feasible and alleviates pain. In addition, these results confirm that ALP, rather than PSA, seems to be a good marker for treatment response. Therefore, Ra-223 may be taken into consideration as part of multimodal treatment for advanced PCa in carefully selected patients.

## MATERIALS AND METHODS

### Patient population and surgical procedure

Ten patients with histologically confirmed, hormone-sensitive prostate cancer (HSPC) and radiological or scintigraphic evidence of ≥2 bone metastases were assessed. Patients underwent RP at the Department of Urology, Ludwig-Maximilians-University, Munich between September 2012 and March 2015. RP was performed with cytoreductive intent on the basis of an experimental oncologic rationale as described previously [12, 13]. Bilateral pLND was performed simultaneously. ADT was initiated after RP and before Ra-223 treatment, and was given continuously. ADT included luteinizing hormone-releasing hormone agonist ± bicalutamide (Table 2). The study was approved by the local ethics committee (UE-Nr-158-16).

### Study design and Ra-223 treatment

In this pilot feasibility study, the following inclusion and exclusion criteria needed to be met: Prior to the first cycle of Ra-223, BS and CT of the abdomen and thorax were performed to confirm bone involvement and to exclude visceral metastases. Only patients without lymph nodes >3 cm in short-axis diameter and visceral metastases on CT were included. Patients after systemic chemotherapy, radionuclide therapy or external beam radiotherapy were excluded. Ra-223 treatment was initiated if hemoglobin level was >10 g/dL, absolute neutrophil count ≥1.5 g/L, and thrombocytes ≥100 g/L. Ra-223 (Xofigo^®^) was acquired from Bayer Healthcare AG (Leverkusen, Germany). Patients were administered intravenous Ra-223 at 50 kBq/kg body weight every 4 weeks (cumulative activities per patient are listed in Table 2). The follow-up visit was scheduled 6-8 weeks after the last administration of Ra-223. At each visit, absolute blood count (hemoglobin, erythrocytes, leukocytes, thrombocytes, neutrophils and lymphocytes) was evaluated to assess bone marrow function and classified using the Common Terminology Criteria for Adverse Events (CTCAE; version 4.0 [22]). Treatment was continued as long as absolute blood count was ≥1.0 g/L for neutrophils and ≥50 g/L for thrombocytes. Otherwise, the decision to discontinue Ra-223 in a patient was based on the treating physicians’ discretion and included newly diagnosed visceral metastases during Ra-223 treatment. ALP and PSA were acquired prior cycle 1, 4, 6 and at follow-up or discontinuation visit. Written informed consent was obtained from each participant before start of Ra-223 treatment.

### Clinical assessment

Mean bone pain intensity, using the four items measuring pain intensity (worst and least pain in the last 24 hours, average pain and pain right now) from the self-reporting Brief Pain Inventory (BPI) questionnaire was recorded before, during and at the end of therapy [15]. Patients were asked to report on bone pain only. Treatment-related adverse events were assessed at each visit.

### Bone scintigraphy

BS was performed at baseline, 1 month after three and 6-8 weeks after completion of all six cycles. In brief, 632 ± 22.5 MBq 99mTc-phosphonates (TECEOS, CIS bio GmbH, Berlin, Germany) were injected intravenously. Whole-body scans were performed using a dedicated gamma camera (Siemens Symbia Intevo 16/6/2; 2014; Siemens Healthcare, Erlangen, Germany). Routine anterior and posterior whole-body images were obtained 3–4 h after injection (256×1024 matrix; scanning speed 15 cm/min). Single Photon Emission Computed Tomography (SPECT)/CT imaging of the thorax, abdomen and pelvis was performed subsequently to better characterize the presence, location and extent of disease (180° circular orbit, 64 steps, 128×128 matrix, and 20s/step). CT was performed as low-dose CT for attenuation correction (130 kV, 20mAS, pitch 1.5, CT slices 5 mm). A dedicated software package was used for image reading (Hermes Hybrid Viewer, version 2.0; Hermes Medical Solutions, Stockholm, Sweden). Images were interpreted by consensus of two experienced nuclear medicine physicians, who were blinded to PSA and ALP levels, and pain scores. BS was scored according to EOD classification [19].

### Statistical analysis

Changes (mean ± standard deviation) in PSA, ALP, and BPI score, as well as absolute and relative changes in blood count from baseline were assessed. Additionally, we classified ALP and PSA response to Ra-223 treatment in each patient.

## Abbreviations

ADT: androgen deprivation therapy
ALP: alkaline phosphatase
BPI: Brief Pain Inventory
BS: bone scintigraphy
CT: computed tomography
EOD: extent of disease
MBq: Megabecquerel
mCRPC: metastatic castration-resistant prostate cancer
mHSPC: metastatic hormone-sensitive prostate cancer
PCa: prostate cancer
PET: positron emission tomography
pLND: pelvic lymph node dissection
QOL: quality of life
PSA: prostate-specific antigen
Ra-223: radium-223 dichloride
RP: radical prostatectomy
SPECT: single photon emission computed tomography
SRE: skeletal-related event
